# Lack of a 5.9 kDa Peptide C-Terminal Fragment of Fibrinogen α Chain Precedes Fibrosis Progression in Patients with Liver Disease

**DOI:** 10.1371/journal.pone.0109254

**Published:** 2014-10-02

**Authors:** Santiago Marfà, Gonzalo Crespo, Vedrana Reichenbach, Xavier Forns, Gregori Casals, Manuel Morales-Ruiz, Miquel Navasa, Wladimiro Jiménez

**Affiliations:** 1 Biochemistry and Molecular Genetics Service, Centro de Investigación Biomédica en Red de Enfermedades Hepáticas y Digestivas (CIBEREHD), Hospital Clínic, Institut d’Investigacions Biomèdiques August Pi i Sunyer (IDIBAPS), University of Barcelona, Barcelona, Spain; 2 Liver Unit, Centro de Investigación Biomédica en Red de Enfermedades Hepáticas y Digestivas (CIBEREHD), Hospital Clínic, Institut d’Investigacions Biomèdiques August Pi i Sunyer (IDIBAPS), University of Barcelona, Barcelona, Spain; 3 Departament de Ciencies Fisiologiques I, Centro de Investigación Biomédica en Red de Enfermedades Hepáticas y Digestivas (CIBEREHD), Hospital Clínic, Institut d’Investigacions Biomèdiques August Pi i Sunyer (IDIBAPS), University of Barcelona, Barcelona, Spain; Saint Louis University, United States of America

## Abstract

Early detection of fibrosis progression is of major relevance for the diagnosis and management of patients with liver disease. This study was designed to find non-invasive biomarkers for fibrosis in a clinical context where this process occurs rapidly, HCV-positive patients who underwent liver transplantation (LT). We analyzed 93 LT patients with HCV recurrence, 41 non-LT patients with liver disease showing a fibrosis stage F≥1 and 9 patients without HCV recurrence who received antiviral treatment before LT, as control group. Blood obtained from 16 healthy subjects was also analyzed. Serum samples were fractionated by ion exchange chromatography and their proteomic profile was analyzed by SELDI-TOF-MS. Characterization of the peptide of interest was performed by ion chromatography and electrophoresis, followed by tandem mass spectrometry identification. Marked differences were observed between the serum proteome profile of LT patients with early fibrosis recurrence and non-recurrent LT patients. A robust peak intensity located at 5905 m/z was the distinguishing feature of non-recurrent LT patients. However, the same peak was barely detected in recurrent LT patients. Similar results were found when comparing samples of healthy subjects with those of non-LT fibrotic patients, indicating that our findings were not related to either LT or HCV infection. Using tandem mass-spectrometry, we identified the protein peak as a C-terminal fragment of the fibrinogen α chain. Cell culture experiments demonstrated that TGF-β reduces α-fibrinogen mRNA expression and 5905 m/z peak intensity in HepG2 cells, suggesting that TGF-β activity regulates the circulating levels of this protein fragment. In conclusion, we identified a 5.9 kDa C-terminal fragment of the fibrinogen α chain as an early serum biomarker of fibrogenic processes in patients with liver disease.

## Introduction

Early detection of fibrosis progression and the development of portal hypertension is of major relevance in the prognosis and treatment of patients with chronic liver disease [1]. Indeed, early recognition of subjects prone to develop these alterations may allow prompt initiation of therapeutic interventions. Therefore, identification of noninvasive biomarkers related to the activation of the fibrogenic process is of major relevance, particularly in those subjects with sustained liver injury [2]. However, despite the numerous attempts to uncover such molecules, this objective has resulted elusive. This is likely related to the natural history of liver disease. With the exception of fulminant hepatic failure, liver disease is an insidious process in which clinical detection and symptoms of hepatic decompensation may occur weeks, months or many years after the onset of injury, and healing may occur without clinical detection [3]. However, in particular clinical circumstances, i.e. patients infected with the hepatitis C virus (HCV), submitted to liver transplantation (LT), it is possible to expect recurrence of hepatic fibrosis and portal hypertension to occur within a short period of time [4]. Thus, these patients constitute a population particularly suitable to identify noninvasive markers of early fibrogenesis.

The current investigation took advantage of the faster development of hepatic fibrosis in HCV-positive LT patients. Serum samples were collected shortly after LT and high-throughput proteomic techniques were used to ascertain whether the proteomic pattern of these samples differs from the proteomic pattern expression obtained from serum samples of non-infected LT patients. Ultimately, the investigation was aimed to identify early circulating serum biomarkers of active fibrogenesis in patients with liver disease.

## Materials and Methods

### Patients

One hundred and nineteen patients admitted to the Liver Unit to undergo a liver biopsy from June 2001 to January 2006 were prospectively considered for this study. Exclusion criteria were presence of ascites, chronic kidney failure in hemodyalisis and moderate or severe acute graft rejection during the first three months, biliary complications or antiviral treatment during the first year after LT in the case of LT recipients. In addition 16 healthy volunteers were also included in the study.

The design of the study was two folded: first we assessed whether the serum proteomic profile of recurrent HCV-LT patients differs from that of non-recurrent HCV-LT patients. The serum proteomic profile and routine liver and renal function tests were initially analyzed in a training set of 10 HCV-RNA recurrent LT patients 6 months post LT that showed a fibrosis stage F≥1 at 1 year after LT. Paired hepatic venous pressure gradient (HVPG) determination was also available in 7 of these patients. The control group consisted in 9 patients without HCV-RNA recurrence, who underwent antiviral treatment before LT and achieve sustained virological response. In addition, serum samples were also collected from 41 non-LT patients with advanced liver disease. The HCV or hepatitis B virus (HBV) was present in 8 and 3 of these patients, respectively, whereas the etiology of liver disease was other than viral in the remaining (9 nonalcoholic steatohepatitis, NASH; 10 alcoholic liver disease, ALD; 4 autoimmune hepatitis, AH; and 7 cryptogenic). Thereafter, the results were validated in a test set of 83 HCV recurrent LT patients. Serum samples were also collected 6 months post-transplantation and the proteomic profile was evaluated along with liver and renal function tests. HVPG measurement in 53 of these patients was also performed.

### Liver Biopsies and paired HVPG measurements

Percutaneous and transjugular liver biopsies and HVPG measurements were performed as we have previously described [5]. Fibrosis stage was scored using the Scheuer classification: no fibrosis (F0), minimal portal fibrosis (F1), periportal fibrosis (F2), fibrosis beyond the portal tract making septums (F3) and cirrhosis (F4) [6].

### Serum fractionation

See (Data S1).

### High-throughput proteomic processing of serum samples

Protein profiling was performed by surface-enhanced laser desorption/ionization time-of-flight mass spectrometry (SELDI-TOF-MS) using the eight-spot format CM10 (weak cationic exchange) ProteinChip arrays (Bio-Rad). In a preliminary study performed to set up the experimental conditions, 2 pooled serum samples from the 9 patients without HCV-RNA recurrence and the 10 patients included in the training set were loaded onto three different types of Protein Chip arrays: H50 (that binds proteins through reverse phase or hydrophobic interactions), CM10 (negatively charged surface that acts as a weak cation-exchanger) and IMAC-30 (Immobilized Metal Affinity Capture surface preactivated with copper). The resulting spectra from each pool were compared and the CM10 array showed the highest number of peaks detected and the highest total signal intensity compared to H50 and IMAC-30; therefore only the CM10 array was used in the subsequent studies. Prior to sample loading, spots were equilibrated two times with 200 µl of CM binding/washing buffer (0.1 M sodium acetate, pH 4). Each sample was loaded in duplicate randomly in order to minimize any systematic error. Forty microliters of fractionated serum sample was incubated in 60 µL of CM binding buffer for 30 minutes on a shaker at room temperature. Afterwards, arrays were washed three times with 200 µL CM washing buffer for 5 minutes at room temperature. Unbound serum proteins were removed by washing twice with deionized water. Thereafter, arrays were air-dried and 1 µL of energy-absorbing matrix (saturated sinapinic acid in an aqueous solution containing 50% acetonitrile and 0.5% TFA) was added twice to each spot. The surface was allowed to air dry between each application. The array was read by using the ProteinChip PBS II reader (BioRad). Each spot was read at low (2500 nJ), medium (3000 nJ) and high (3500 nJ) energy laser intensities. The mass-to-charge ratio (m/z) was set from 1.000 to 25.000 m/z for the low-energy laser intensity, between 2.500 and 200.000 m/z for the medium-energy laser intensity and from 5.000 to 200.000 for the high-energy laser intensity. All spectra were calibrated using two external calibration standards (all-in-one peptide standard and all-in-one protein standard, BioRad). A peak resolution was optimized within 5.000 m/z, 12.000 m/z or 19.000 m/z according to low, medium or high energy laser intensity, respectively.

### Data acquisition and analysis

All data were processed with the ProteinChip Data Manager Client 4.1 software (Bio-Rad). To minimize the possible random error and spectral outliers, all the raw data was normalized by the average total ion current across the group and all spectra differing by twice the standard deviation or more from the mean were deleted. Furthermore, the baseline was also corrected by adjusting the parameter to 30 times the expected peak width. For the peak selection, several parameters were selected for the identification of peak clusters. Thus, only peaks with a signal to noise equal or greater than 5; with a valley depth superior than three; found in a minimum of 20% of all spectra and with an m/z error below the 0.3% for the low-energy laser intensity spectra and below 2% for the medium- and high-energy laser intensity spectra, were considered. Subsequently, all peak clusters detected were verified manually. Relabeling, removal or addition of peaks was performed when necessary. To test the quality of the assay, pooled normal sera from two individuals was assessed. Five protein peaks randomly selected over the course of the study were used to calculate the coefficient of variance (CV) as described [7]. We then determined the reproducibility of the SELDI spectra, both within and between arrays (intra-assay and inter-assay, respectively). The intra-assay (spot to spot) CV was 11.95% for peak intensity and 0.02% for mass accuracy. The inter-assay (chip to chip) CV was 21.96% for peak intensity and 0.03% for mass accuracy.

### Identification of candidate biomarker

See (Data S2).

### Cell Culture

HepG2 cells were obtained from American Type Culture Collection (ATCC, Manassas, VA, USA). This immortalized, stable cell line can be repeatedly frozen, thawed and propagated. HepG2 cells were seeded (2×10^6^ cells/well) in vented T-75 flasks and grown to confluence in Dulbecco’s Modified Eagle Medium (DMEM), supplemented with 50 U/ml penicillin, 50 µg/ml streptomycin and 10% of fetal calf serum (FCS). Thereafter, cells were switched to 1% FCS and incubated (37°C) under normoxic (21% O_2_, 5% CO_2_) or hypoxic conditions (5% O_2_, 5% CO_2_) in a controlled O_2_ water-jacketed CO_2_ incubator (Forma Scientific Series II, 3131, Marietta, OH) or treated with TNF-α (10 ng/ml, Sigma, St Louis, MO), lipopolysaccharide (LPS, 10 ng/ml, Sigma), AII (80 pM, Sigma), Endothelin-1 (2 nM, Sigma), Apelin (100 nM, Phoenix Pharmaceuticals, Burlingane, Ca), Fibronectin (10 ng/ml, Sigma), Interleukin-1β (20 ng/ml, Sigma) and TGF-β (10 ng/ml, R&D Systems, Minneapolis, Mn). All experiments carried out in cell lines were reproduced three times in at least 2 independent assays. Conditioned media were harvested, concentrated (80∶1) using 3000 MW Amicon Ultra centrifugal filters (Millipore Corp) and the presence of the fibrinogen α C-chain was assessed by SELDI-TOF-MS as described above.

### Messenger RNA expression of human fibrinogen α, β, and γ chains in HepG2 cells

See (Data S3).

### Measurements and statistical analysis

The same day of the liver biopsy, 20 ml of blood were obtained in a fasting status. Serum was stored at −80°C, and serum albumin, aspartate aminotransferase (AST), alanine transaminase (ALT), bilirrubin and blood urea nitrogen (BUN) were measured with the ADVIA 2400 Instrument (Siemens Healthcare Diagnostics, Tarrytown, NY, USA). Amino-terminal propeptide of type III procollagen (PIIINP), hyaluronic acid (HA), and tissue inhibitor of matrix metalloproteinase type-1 (TIMP-1) were measured in all patients by a CE-marked random-access automated clinical immunochemistry analyzer that performs magnetic separation enzyme immunoassay tests (ADVIA Centaur, Siemens Healthcare Diagnostics, Tarrytown, NY, USA). The enhanced liver fibrosis (ELF) score was calculated using the algorithm recommended in the CE-marked assay [ELF = 2.278 + 0.851 ln(HA) + 0.751 ln(PIIINP) + 0.394 ln(TIMP-1)] blood tests.

Statistical analysis of the results was performed by the non parametric Mann-Whitney U test and the Kruskal-Wallis test with the Dunn post hoc test as appropriated. Quantitative data were analyzed using GraphPad Prism 5 (GraphPad Software, Inc. San Diego, CA).

### Ethics Statement

We obtained written informed consent from all patients included in the study and the investigation was approved by the Investigation and Ethics Committee of the Hospital Clinic of Barcelona following the ethical guidelines of the 1975 Declaration of Helsinki.

## Results

### Mass Spectrogram of LT patients with early fibrosis recurrence significantly differs from that of non recurrent LT patients

**Figure 1:**
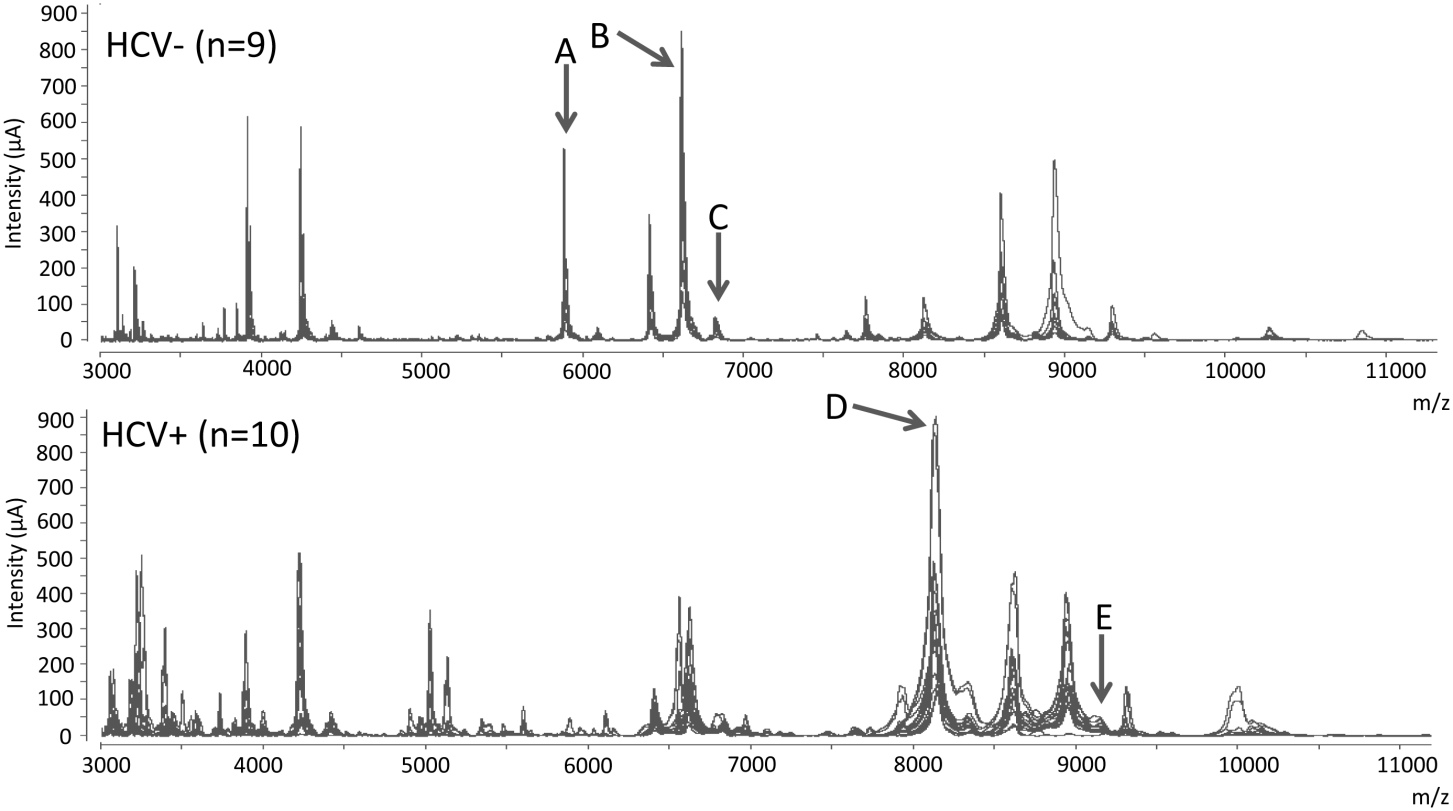
Differential proteomic profile between HCV− and HCV+ patients submitted to LT. Fragment ranging from 3000 to 11000 m/z of the differential SELDI-TOF-MS spectra of all serum samples obtained from patients 6 months after LT. The upper figure shows all overlapped spectra from HCV-negative patients (n = 9). The bottom figure shows the spectra from HCV-infected patients (n = 10). Arrows indicate peaks showing significantly different intensities between HCV− and HCV+ patients. Upper letters correspond to the identification peak noted in table 2.

The principal demographic values of patients included in the definition group are shown in Table 1. As per the selection criteria, most recurrent HCV patients showed higher fibrosis and ELF scores, elevated HVPG measures and greater AST and ALT values than non recurrent HCV subjects. Figure 1 depicts a portion of the spectra of all the samples investigated in this training group, ranging between 3000 and 11000 Daltons; the mass to charge ratio analyzed. The expression pattern of the spectrograms obtained from non recurrent HCV patients clearly differed from those of recurrent HCV subjects. Six statistically different peaks, identified in the figure as peptides A, B, C, D, and E, were detected. As shown in Table 2 the signal intensity of four of these peaks (A, B, C, and F) was markedly higher in non recurrent than in recurrent patients whereas an inverse situation was observed in the remaining two peaks (D and E). The most remarkable difference was detected on analyzing peptide A (5905 m/z), since it was fully evident in all samples obtained from non recurrent HCV patients but almost suppressed in the serum of recurrent HCV individuals.

**Table 1:** Baseline characteristics of liver transplant recipients in non recurrent and recurrent hepatitis C patients.

	NON RECURRENTHCV SUBJECTS (n = 9)	RECURRENT HCVSUBJECTS (n = 10)	P*
Sex (M/F)	7/2	3/7	
Age (yr)	53.9±3.3	55.9±2.3	NS
Fibrosis score (1 yr after LT)			
F 1–2 (n)	-	4	
F 3–4 (n)	-	6	
HVPG (1 yr after LT)			
HVPG<6 mm Hg (n)	-	0	
HVPG 6–10 mm Hg (n)	-	2	
HVPG≥10 mm Hg (n)	-	5	
Bilirubin (mg/dl)	0.9±0.1	1.3±0.3	NS
Albumin (g/l)	43.1±1.1	37.8±2.1	<0.05
BUN (mg/dl)	27.4±3.5	34.0±3.4	NS
AST (U/l)	28.9±4.5	152.3±47.1	<0.001
ALT (U/l)	37.7±6.6	202.7±60.9	<0.001
Total proteins (g/l)	67.9±1.6	63.1±3.3	NS
PT(%)	93.2±2.4	86.3±3.4	NS
ELF score	9.9±0.3	11.7±0.5	<0.05

*in comparison to non recurrent HCV subjects (Mann-Whitney U test), NS: non significant. Results are given as mean±SEM.

**Table 2:** Intensity values (µA) of peaks showing different patterns of expression in the two groups of patients.

PEPTIDE	m/z (Da)	NON RECURRENTHCV SUBJECTS (n = 9)	RECURRENT HCVSUBJECTS (n = 10)
A	5905	257.1±43	5.99±3.49^d^
B	6639	687.4±96.3	176.8±32.5^c^
C	6845	71.9±8.9	27.13±5.42^b^
D	8144	76.9±22.5	392.8±64.8^d^
E	9172	15.1±6.4	26.03±3.37^a^
F	12986	6.6±1.2	1.99±0.41^a^

a p<0.05, ^b^p<0.01, ^c^p<0.001, ^d^p<0.0001; in comparison to non recurrent HCV subjects (Mann-Whitney U test). Results are given as mean±SEM.

### Neither LT nor HCV infection account for Mass Spectrogram differences in patients with active fibrogenesis

**Figure 2:**
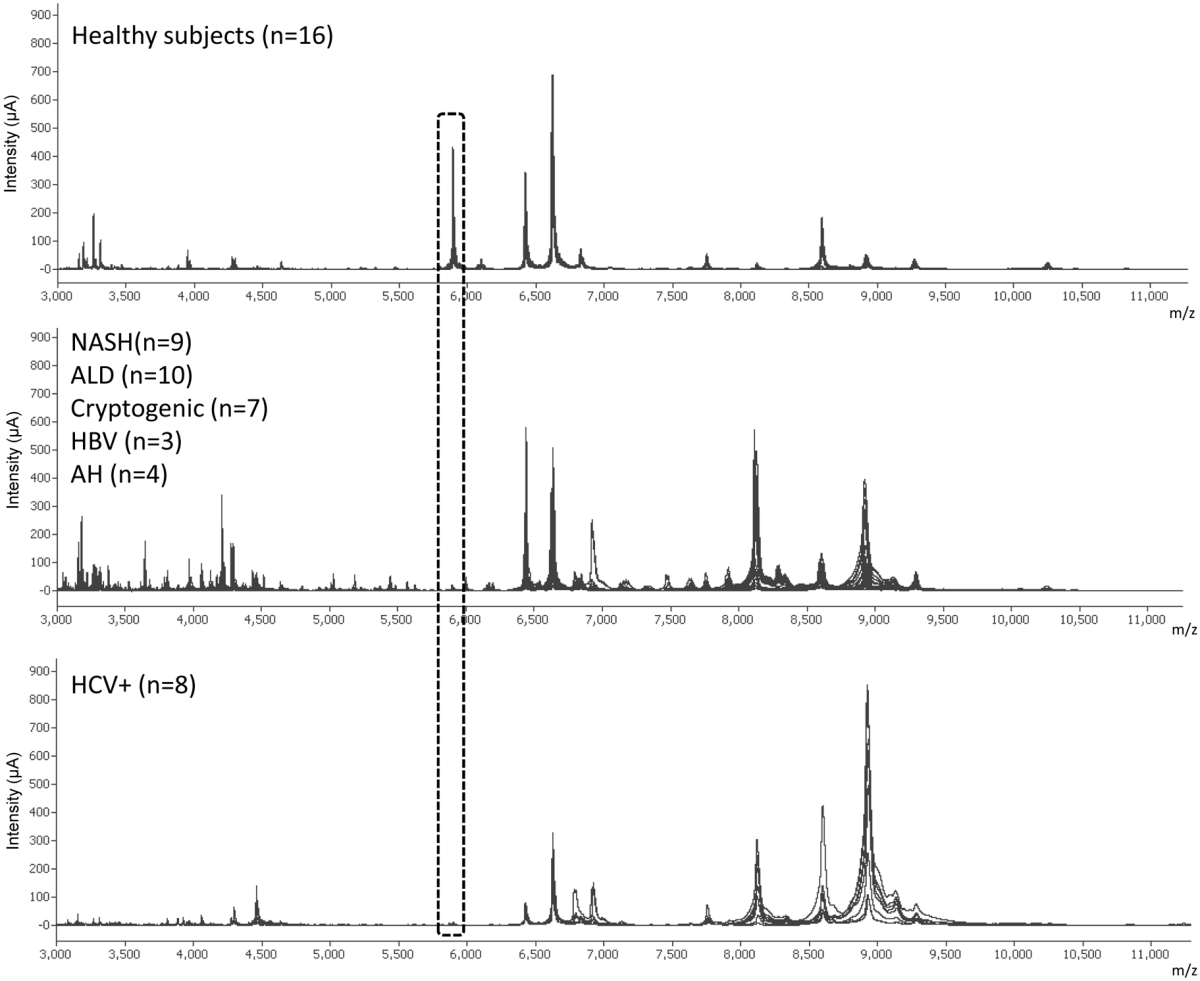
Lack of the 5.9 kDa protein peak in non-LT fibrotic patients. Portion of the SELDI-TOF-MS spectra comprised between 3000 and 11000 m/z of serum samples obtained from healthy subjects (n = 16) and patients with liver fibrosis of several etiologies (NASH, ALD, cryptogenic, AH, HBV and HCV). The lack of the protein peak at m/z 5905 is clearly associated with fibrogenesis regardless of its etiology.

An intriguing question arising from the above described results was to elucidate whether these findings result from the particular characteristics of HCV transplanted patients rather than to a differential feature characterizing early fibrogenic processes. Thus, the serum proteomic spectrum was next analyzed in healthy individuals, in non-LT fibrotic HCV infected patients and in non HCV fibrotic patients. The clinical and demographic characteristics of these subjects are shown in table 3. Fibrosis scores and liver function tests of the two groups of fibrotic patients were similar to those obtained in HCV LT patients. The mass spectrograms of all individuals included in this portion of the investigation are shown in figure 2. The upper, middle and lower parts correspond to healthy subjects, fibrotic non HCV subjects and fibrotic HCV patients, respectively. Since in the previous experiments the most striking differences were observed with peptide A, in this and the subsequent experiments we focused on the peak with a mass/charge ratio of 5905 Da. All serum samples analyzed from healthy subjects showed a spectrogram compatible with the presence of this peptide whereas pathological serum samples, either from fibrotic non LT HCV patients or fibrogenic non HCV subjects showed the absence of this peptide or very low intensity peaks. These results indicate that neither HCV nor LT account for the suppressed expression of the A peptide in serum samples of fibrotic patients.

**Table 3:** Baseline characteristics of healthy subjects and HCV-infected and non-infected fibrotic patients.

	HEALTHY SUBJECTS (n = 16)	HCV INFECTED PATIENTS (n = 8)	NON INFECTED FIBROTIC PATIENTS (n = 33)
Sex (M/F)	(9/7)	(5/3)	(19/14)
Age (yr)	35.6±2.5	48.7±3.6	52.3±2.4***
Fibrosis score			
F 1–2 (n)	-	2	11
F 3–4 (n)	-	6	22
Etiology			
HCV (n)	-	8	-
HBV (n)	-	-	3
NASH (n)	-	-	9
ALD (n)	-	-	10
AH (n)	-	-	4
Cryptogenic (n)	-	-	7
Bilirubin (mg/dl)	0.6±0.1	0.5±0.06	1.2±0.2* ^,b^
Albumin (g/l)	41.0±1.5	42.9±0.6	40.9±1.2
BUN (mg/dl)	15.2±2.1	14.4±1.6	18.1±2.2
AST (U/l)	20.9±1.6	64.0±7.9***	56.3±6.6***
ALT (U/l)	15.9±2.3	82.1±8.8***	79.1±15.4***
Total proteins (g/l)	71.3±1.2	78.6±2.3	75.4±1.7
PT(%)	96.5±0.9	89.8±3.1	86.4±2.4*
ELF score	8.6±0.4	9.6±0.6	9.7±0.2
INR	1.00±0.01	1.04±0.03	1.10±0.03

*p<0.05, ***p<0.001 in comparison to healthy subjects and ^b^p<0.01 in comparison to HCV infected patients (Kruskal-Wallis test with the Dunn pos hoc test). Results are given as mean±SEM.

### The Mass Spectrogram of most HCV infected patients lacks a 5.9 kDa fragment

**Figure 3:**
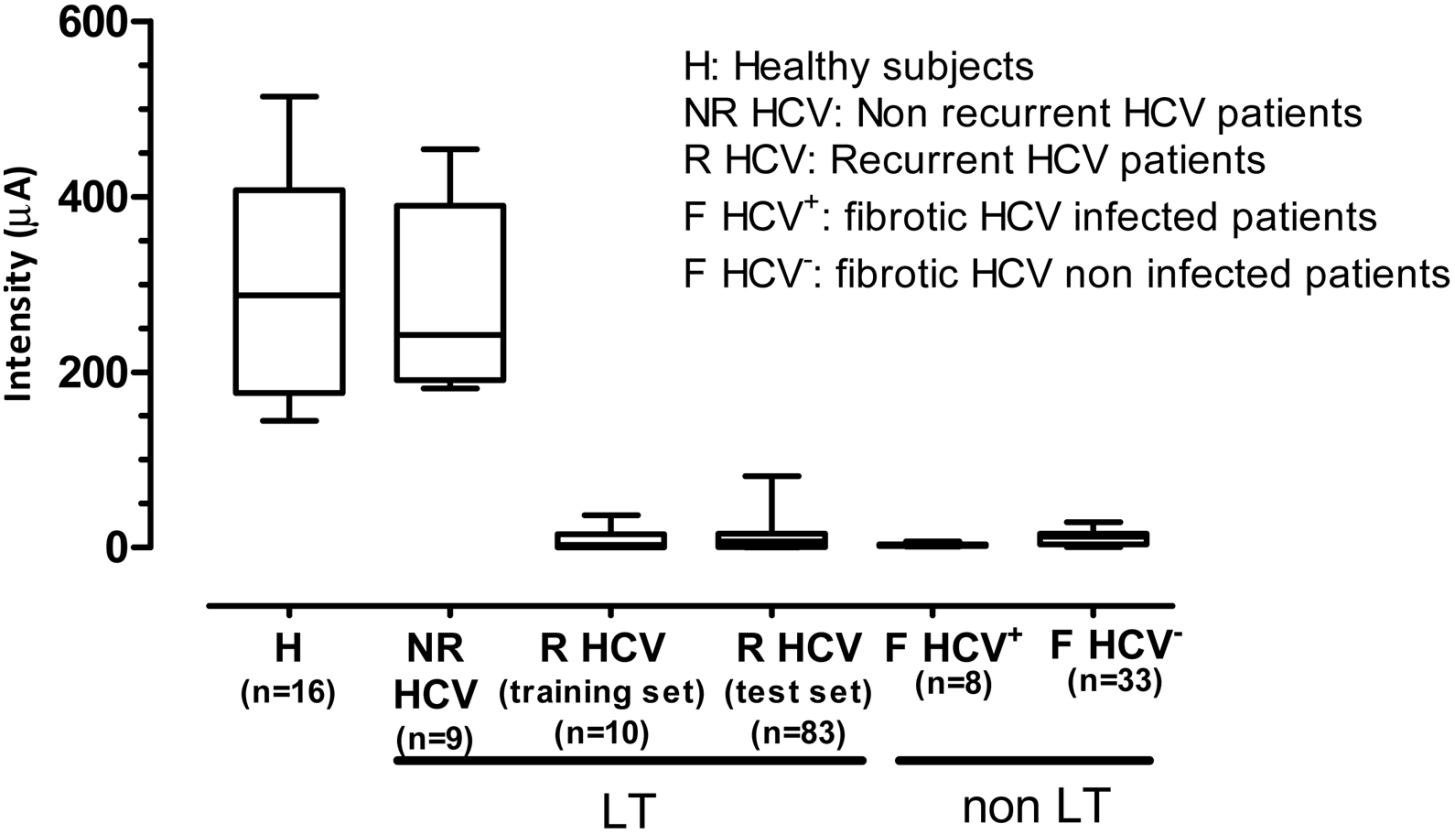
Comparison of the 5.9 kDa protein peak intensity between all the groups analyzed. SELDI-TOF-MS intensity values of the 5.9 kDa peak of the different groups of patients studied. Intensity of the peak was markedly suppressed in all patients under an active fibrogenic process.

To further confirm that peptide A behaves as an early serum biomarker of fibrosis, mass spectrometric analysis was performed in a test group of serum samples obtained from 83 HCV recurrent patients 6 months after LT. HVPG was assessed in 53 of these patients and the average value was of 5.5±0.8 mm Hg. All the serum samples showed a quite similar expression pattern and coincidences included both the different peptide fragments detected and the signal intensity of these fragments (Data S4). The most relevant finding was, however, that the mass spectrum corresponding to peptide A showed a very low peak intensity in all samples obtained from HCV recurrent patients. In fact, in 53 out of the 83 patients the peak intensity at m/z 5905 was under the background levels of 10 µA and in the remaining samples, intensities ranged between 11.5 and 81.4 µA (figure 3). Thus, these results confirm the findings obtained in the training group in a larger group of subjects.

### The 5.9 kDa protein is a C-terminal fragment of the fibrinogen α chain

**Figure 4:**
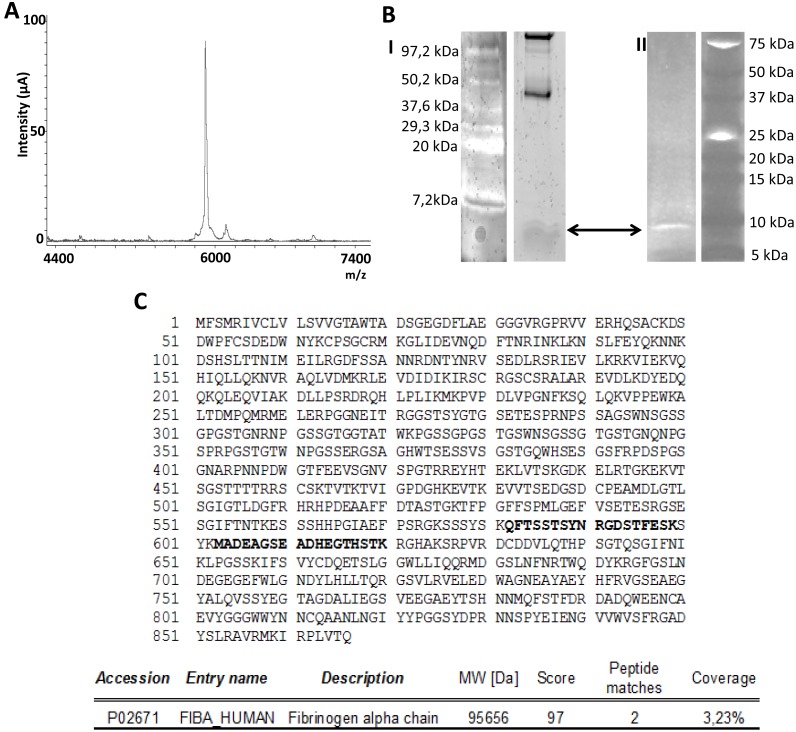
Isolation, separation and identification of the 5.9 kDa protein peak. A/ Spectra obtained by SELDI-TOF-MS showing the isolated protein peak after the purification process. B/ The isolated band indicated with arrows, after running two tris-tricine gels, one stained with sypro (I) and the other with oriole (II) staining. C/ Fragment of the Fibrinogen α-Chain identified as the differential protein peak by the amino acid sequencing. The two sequences identified are shown in bold.

To isolate the protein of interest and to determine candidate protein identity, serum samples from two healthy subjects containing high SELDI intensity were pooled and separated by tricine-SDS-PAGE (figure 4B). The band at 5.9 kDa was excised trypsinized and analyzed by LC-MS/MS. As shown in figure 4C, two peptide sequences were identified, which matched with the human fibrinogen α C-chain at 3.23% coverage, suggesting that suppression of the fibrinogen α C-chain 5.9 kDa fragment is an early surrogate indicative of active fibrogenesis in patients with liver disease.

### TGF-β reduces the expression of the fibrinogen α chain but not of the β and γ chain in HepG2 cells assays

**Figure 5:**
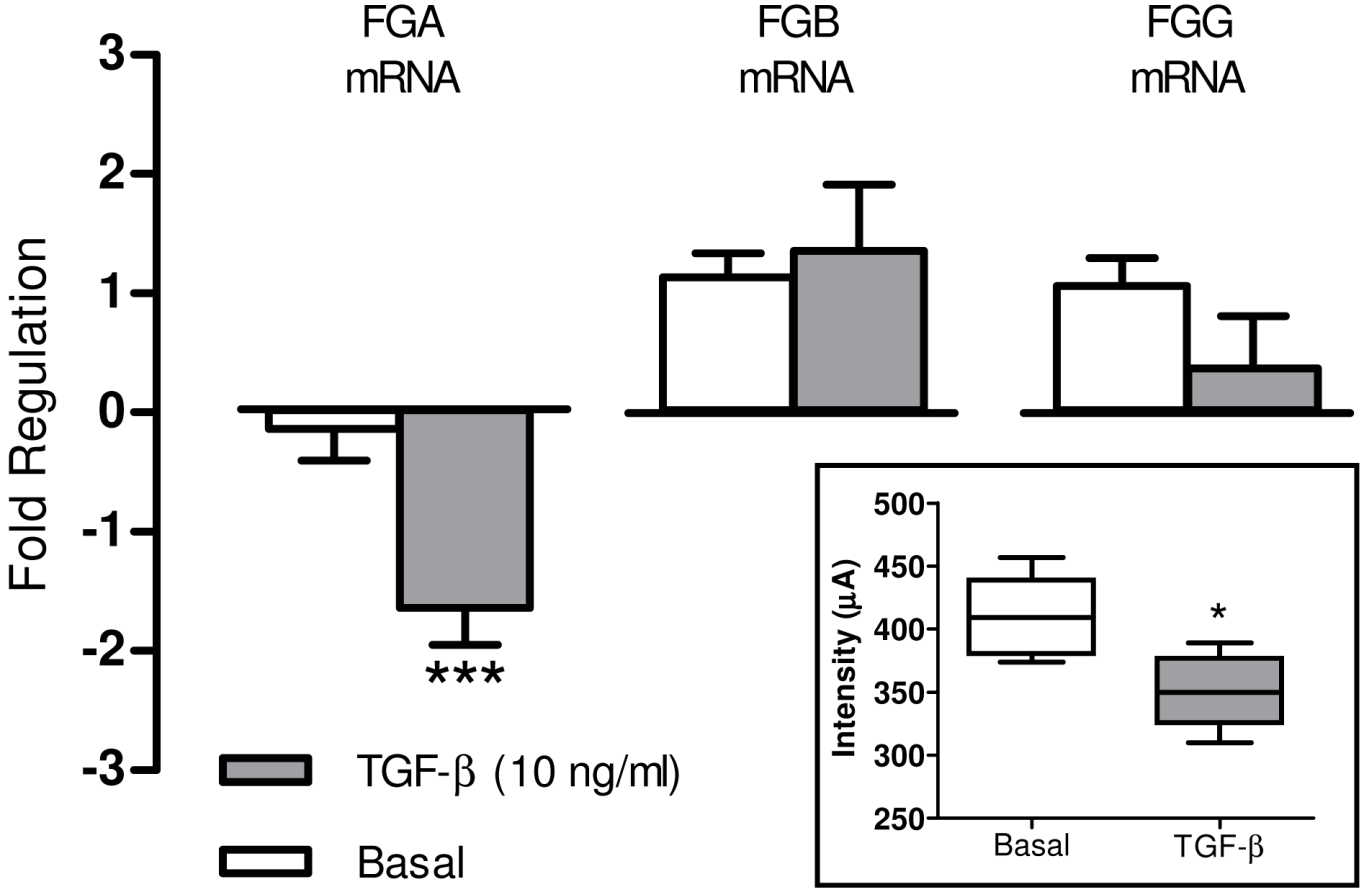
TGF-β reduces the fibrinogen α-chain expression in HepG2 cells. Fold regulation in Fibrinogen Alpha Chain (FGA), Beta Chain (FGB) and Gamma Chain (FGG) genes regulation in HepG2 cells after 6 hours of treatment with TGF-β (10 ng/ml). Results are given as mean ± SE; ***p<0.001 vs. basal. Statistical analysis was calculated by unpaired Student’s t test. The insert shows the intensity values of the 5.9 kDa peak detected in the cellular supernatant of HepG2 cells after 48 hours of treatment with TGF-β (10 ng/ml). Results are given as mean ± SE; *p<0.05 vs. basal. Statistical analysis was calculated by Mann-Whitney U test.

To unveil potential mechanisms governing the release of the 5.9 kDa peptide C-terminal fragment of the fribrinogen α chain in the serum of patients under an early fibrogenic process, HepG2 cells were treated with well known proinflammatory stimuli (TNF-α, LPS and Il-1β) or profibrogenic substances (AII, ET-1, Apelin, Fibronectin and TGF-β) for 6 hours. With the exception of TGF-β, none of these substances induced significant changes in the expression of human fibrinogen chains messenger. However, TGF-β markedly reduced the expression of the α chain in cultured human hepatocytes. As shown in figure 5, this phenomenon was specific for the α chain since no significant changes were observed on analyzing the β and γ chain messengers. In addition, culture media of cells treated with TGF-β displayed diminished abundance of the 5.9 kDa fragment of the fibrinogen α C-chain in comparison to untreated cells.

## Discussion

Evaluation of the extension and aggressiveness of the fibrogenic process in the injured liver is of major relevance for the diagnosis, prognosis and treatment of patients with hepatic disease [8]. The methods currently available to assess liver fibrosis include the serological determination of several parameters related to liver function and hepatic remodeling, imaging techniques, such as Fibroscan or ARFI and the use of invasive procedures such as HVPG measurement or liver biopsy, the latter still being the most widely accepted gold standard method for assessing liver fibrosis [9]. The specific limitations of each of these methods have been extensively discussed previously [10]. The risk of complications and low sensitivity for mild or moderate fibrosis are among the most remarkable limitation for invasive and non invasive methods, respectively [11]. Recently, a liver fibrosis score, namely ELF, which combines the serum concentrations of substances related to collagen metabolism (PIIINP) and tissue remodeling (TIMP-1 and HA), has progressively been incorporated among the most common diagnostic tools to evaluate liver fibrosis. However, whereas this technique was found to be highly accurate in patients with advanced fibrosis (F3–F4 stage) [12]–[14] it appeared to be less efficacious in the diagnosis of mild or moderate fibrosis (F1–F2 stage) [15], [16]. Early detection of active fibrogenic activity, therefore, still remains an open challenge in liver disease.

Fibrosis progression evolves over long periods of time, with this representing one of the most relevant difficulties to identify specific early biomarkers of fibrosis. In the current investigation this issue was overcome by assessing the proteomic profile of HCV-positive LT recipients in a training set of serum samples. Blood samples were obtained at 6 months after LT and a liver biopsy was performed 1 year after surgery to define fibrosis stage. It is well known that fibrosis progression is accelerated in recurrent hepatitis C, with 15% to 47% of LT recipients developing fibrosis/cirrhosis within the first 3 years post transplantation [17]. Therefore, rapid fibrosis progression is a major characteristic of this group of patients and for this reason they are particularly suitable to uncover serum tags of hepatic fibrosis.

SELDI-TOF-MS technology or protein chip profiling combines mass spectrometry with a surface enhanced biochip which allows uniform and reproducible binding and desorption of biomarkers [18]. SELDI-TOF-MS also incorporates sample prefractionation. This markedly decreases the complexity of protein rich fluids, such as serum, allowing comparison of peak intensity between samples using large sample sets [19]. In the current study, serum proteins were fractionated by anion exchange chromatography based on their isoelectric points using a pH gradient. The resulting fraction was bound to a weak cation exchange surface to create an array of Protein Chip spots. This surface was selected according to its higher accuracy and reproducibility yields. Using this technology we were able to simultaneously detect relative protein expression levels over a range of molecular masses of 2 to 180 kDa, although the 2–20 kDa range appeared to be the most sensitive. By means of this profiling system, we found at least 6 serum biomarkers that were differentially increased or decreased in recurrent HCV patients. Among them, a protein of 5.9 kDa (protein A) was fully suppressed in the serum of all the HCV patients included in the training set. In contrast, readily detectable levels of this protein were detected in all non-recurrent HCV patients. We assessed whether LT and/or HCV infection account for the different expression patterns of peak A in serum samples of non-transplanted HCV positive and HCV negative subjects with fibrosis. The demographic and biochemical characteristics of patients with fibrosis included in the training set of samples were quite similar to those displayed by the fibrotic patients of this protocol with the exception of hepatic enzymes which, as expected, were higher in LT recurrent HCV patients than in fibrotic non-transplant patients. Both, the proteomic profile of the HCV positive samples and the proteomic profile of fibrotic patients non-infected sera, showed no or very low intensity peaks at the 5.9 kDa spectra. This markedly differed from the proteomic analysis of the serum samples of healthy subjects included in this set of experiments because all displayed consistent amounts of protein A. Interestingly, different etiologies (NASH, ALD, HBV, AH, cryptogenic) accounted for liver fibrosis in negative HCV patients, further emphasizing the close relationship between the lack of the 5.9 kDa protein and the fibrogenic process.

Next, the spectral data obtained in the test set were applied for validation purposes. All serum samples included in the test set showed an intensity m/z 5905 peak well below the values found in both healthy subjects and non recurrent HCV patients. Indeed, in most of these samples the A peak was not detected (figure 3). Overall, our results showing markedly decreased expression of the m/z 5905 in the spectral profile of all samples from patients with fibrosis further strengthen the highly sensitive diagnostic performance of this peak.

A major limitation of SELDI-TOF-MS technology is related to the unfeasibility of directly identifying the protein of interest. In fact, for the majority of protein identifications it is necessary to achieve the enrichment of the specific peak by chromatography procedures and purification by SDS gel electrophoresis with subsequent triptic digest. In our investigation, amino acid sequencing of the trypsin digest of the 5.9 kDa protein revealed it to be a fragment of the fibrinogen α C-chain. Human fibrinogen is a circulating 340 kDa glycoprotein which has been shown to be of hepatic origin *in vivo*. Moreover, inflammatory stimuli may induce *in vitro* secretion of this glycoprotein in non hepatic cells including epithelial cells, granulosa cells, cervical carcinoma cells and trophoblasts [20]. However, current evidence strongly suggests that the largest site of human plasma fibrinogen is the hepatocytes [21]. It is comprised of two symmetric half molecules bound by a disulphide knot, each consisting in one set of three different polypeptide termed Aα, Bβ and γ. Each of these polypeptides is encoded by a separate gene located on chromosome four. The predominant Aα of circulating fibrinogen contains 610 aa (63.5 kDa), the Bβ chain 461 aa (56 kDa) and the γ chain is heterogeneous, but the most abundant form consists of 411 aa (48 kDa). The protein shows extensive post translational modification including phosphorylation, sulphation, glycosylation and hydroxylation. The fibrinogen α C-domain of the human fibrinogen is the C-terminal two-thirds of the Aα chain that extends from the coiled oil portion of each half of the dimeric fibrinogen molecule [22], [23]. The α C-fragments are released into circulation as natural by-products of fibrinolytic systemic activation and are therefore, found in the systemic circulation in healthy individuals [24]. Our results showing almost suppressed expression of the 5.9 kDa fragment of the α C-chain of fibrinogen in patients undergoing a fibrogenic process are in agreement with those previously reported by Nomura F et al in heavy drinkers [25]. Furthermore, these authors showed that serum levels of this fragment were recovered when alcohol intake has ceased for more than 3 months and they also extended their findings to HCV infected patients [26]. Later, this fragment was described as having diagnostic value in patients with acute respiratory syndrome [27], breast cancer [28] and pancreatic adenocarcinoma [29].

The regulation of total human fibrinogen by a number of proinflammatory agents has been previously investigated using the HepG2 hepatocellular carcinoma cell line [30]. This *in vitro* model faithfully recapitulates fibrinogen expression including α, β and γ fibrinogen [31] and has been used to study fibrinogen production and regulation *in vitro* [32]. Accordingly we subsequently assessed the potential regulatory role of several candidate mediators on α-fibrinogen expression in HepG2 cells. A number of proinflammatory/profibrogenic agents that have previously been involved in the pathogenesis of the fibroproliferative processes [33]–[35] were tested. Among them, only TGF-β showed significant regulatory activity on α-fibrinogen mRNA expression and decreased 5.9 kDa fibrinogen αC-fragment intensity. Of note was, however, that the fold change in the fibrinogen αC-fragment induced by TGF-β in HepG2 cells was makedly lower than that observed in samples from fibrotic patients. The marked differences between the *in vivo* and *in vitro* experimental conditions likely account for this discordance. For instance, HepG2 is a human derived carcinoma cell line that shows altered abundance of TGF-β receptors [36], [37] which in turn could result in some sort of resistance to this cytokine. On the other hand it is well known that regulation of acute-phase proteins is mediated by a combination of cytokines thus raising the possibility that additional factors involved in inflammatory processes also regulate the expression of the 5.9 kDa fragment of fibrinogen [38]. Our results are in line with past studies in which TGF-β inhibited the induction of fibrinogen produced by IL-6 and decreased the synthesis of fibrinogen in HepG2 and HepB cells [38], respectively. These latter experiments also showed a parallel diminution in α-fibrinogen mRNA levels. This phenomenon seems to be mediated by post-transcriptional mechanisms since TGF-β did not modify fibrinogen gene transcription, suggesting that the effect of this cytokine in liver cells is regulated at the level of mRNA stability [39]. Overall, all these results indicate that TGF-β may regulate the synthesis of α-fibrinogen at the postranscriptional level.

In summary, the current investigation took advantage of the faster development of hepatic fibrosis in HCV-positive LT patients to identify early circulating serum biomarkers of active fibrogenesis in patients with liver disease. Using high throughput SELDI-TOF-MS technology we unveiled a differential protein pattern profile between early fibrosis recurrence and non recurrent LT patients. Six protein peaks displaying statistically significant different intensities were observed within a range of 1000 to 25000 m/z. The peak located at 5905 m/z showed the most remarkable difference, since it was fully detected in non-recurrent LT patients but was almost suppressed in recurrent LT patients. Similar results were found when comparing samples of healthy subjects with those of non LT fibrotic patients both HCV positive and negative, indicating that our findings were not related to either LT or HCV infection. Identification of this protein peak showed more than a 99% coincidence with a C-terminal fragment of the fibrinogen α chain. Moreover, cell culture experiments demonstrated that TGF-β downregulates α-fibrinogen mRNA expression and decreases the peak intensity of the m/z 5.9 KDa protein in HepG2 cells. In conclusion, we identified a 5.9 kDa C-terminal fragment of the fibrinogen α chain as a serum biomarker of early fibrogenic processes in patients with liver disease. Since TGF-β inhibited α-fibrinogen mRNA expression in HepG2 cells it is temptative to speculate that the activation of this cytokine in the early phases of liver injury could be responsible for the impairment in the circulating levels of the fibrinogen α C-chain fragment in patients with active hepatic fibrogenesis.

## Supporting Information

Data S1 **Materials and Methods corresponding to the serum fractionation procedure.**
(DOC)

Data S2 **Materials and Methods corresponding to the identification of the candidate biomarker.**
(DOC)

Data S3 **Materials and Methods corresponding to the analysis of the messenger RNA expression of human fibrinogen α, β, and γ chains.**
(DOC)

Data S4 **Spreadsheet containing all protein peaks detected in all the samples included in the study.**
(XLS)
