# Supplementary material for: Lack of a 5.9 kDa Peptide C-Terminal Fragment of Fibrinogen α Chain Precedes Fibrosis Progression in Patients with Liver Disease
Source: PLoS One. 2014 Oct 2;9(10):e109254. doi: 10.1371/journal.pone.0109254 (PMC4183580; doi:10.1371/journal.pone.0109254)
Supplement: Data S1 — Materials and Methods corresponding to the serum fractionation procedure. (DOC) [file pone.0109254.s001.doc]

# EXPANDED EXPERIMENTAL PROCEDURES

***Serum fractionation*:**

The samples were fractionated by pH, using a protein chip serum fractionation kit (ProteinChip Serum Fractionation Kit, Bio-Rad Laboratories, Hercules, CA). The kit consists of a 96 well filtration plate with Q HyperD F anion-exchange beads that required rehydration and equilibration before use. Two hundred microliters of rehydration buffer (50 mM Tris-HCl, pH 9) was added to each well and incubated for 1 hour at room temperature. Thereafter, each well was equilibrated three times with 200 μl of U1 buffer [1 M Urea, 0.2% (w/v) CHAPS, 50 mM Tris-HCl, pH 9]. Prior to fractionation, 20 μL of serum sample was mixed with 30 μL of U9 buffer [9 M Urea, 2% (w/v) CHAPS, 50 mM Tris-HCl, pH 9] in a 96-well V-bottom plate for 20 minutes at 4ºC in a horizontal orbital microplate shaker. The sample was then diluted with 50 μL of U1 buffer. One hundred μl of the diluted serum sample was applied to each well and incubated for 30 min. at room temperature under constant shaking. Flow-through was collected by vacuum filtration into V-bottom microplates. The anion-exchange resin was incubated with an additional 100 μL of Tris HCl buffer (50 mM Tris-HCl, 0,1% OGP, pH 9) for 10 minutes at room temperature with shaking. The wash was collected by vacuum filtration. This procedure was repeated twice with 100 μl each of appropriate buffers with decreasing pH (pH 7, 5, 4 and 3). The final wash was performed with an organic wash buffer containing 33% (vol/vol) isopropanol and 16.7% (vol/vol) acetonitrile in 0.1% trifluoroacetic acid (TFA). The six fractions, 200 μL each, were kept at -80ºC until analysis. Fraction 1 (pH 9) was selected for subsequent analysis. Serum fraction was empirically determined from two pools of serum samples obtained from HCV positive (n=10) and HCV negative (n=9) LT patients according to a protocol aimed to maximize the number of identifiable protein peaks while reducing peak-to-peak interference.
