# Supplementary material for: Lack of a 5.9 kDa Peptide C-Terminal Fragment of Fibrinogen α Chain Precedes Fibrosis Progression in Patients with Liver Disease
Source: PLoS One. 2014 Oct 2;9(10):e109254. doi: 10.1371/journal.pone.0109254 (PMC4183580; doi:10.1371/journal.pone.0109254)
Supplement: Data S2 — Materials and Methods corresponding to the identification of the candidate biomarker. (DOC) [file pone.0109254.s002.doc]

# EXPANDED EXPERIMENTAL PROCEDURES

***Identification of candidate biomarker***:

*Sample purification*: Ion exchange fractionation was undertaken on a Q ceramic HyperD F mini spin column (Bio-Rad) pre-equilibrated with 800 μl of binding washing buffer (50 mM Tris-HCl, pH 9) and 800 μL of U1 buffer (three times), respectively. Serum samples from healthy subjects were diluted at a ratio of 1:1.5 in U9 buffer. Treated samples were diluted 1:1 in binding washing U1 buffer and applied to the spin column followed by incubation for 45 min. at 5ºC. Unbound proteins were eluted by centrifugation. Then 400 μl of elution buffer (50 mM Tris-HCl, 0.1% OGP, pH 9) were applied to the minispin column and centrifuged at 100 g, room temperature for 1 min. The flow-through was also collected. Eight minispin columns were used, and 6.4 mL of the eluted fraction were obtained in total. Eluted fractions containing the candidate biomarker were pooled and concentrated by molecular weight cut-off membrane fractionation (Amicon Ultra-4, 30000 MWCO, Millipore Corp. Billerica, MA). SELDI-TOF-MS was used to monitor the resulting concentrate for the peak of interest. The sample was then further purified on a CM ceramic HyperD F sorbent minispin Column. The column was equilibrated by loading 800 μl of binding buffer (0.1 M sodium acetate, pH 4.0) three times. The concentrated protein sample was mixed at a ratio of 1:1.5 with binding buffer and applied to the column. 375 μL of binding buffer were also added. The column was incubated for 45 min at 5ºC, centrifuged and the eluate was discarded. 750 μl of 0.5 M NaCl were added to the column, incubated for 20 min at room temperature and centrifuged at 100 g for 1 min. Two additional cut-off membrane fractionation steps were performed (Amicon Ultra-4, 3000 MWCO and Amicon Ultra-0.5, 10000 MWCO, Millipore Corp.) to completely isolate the candidate biomarker. SELDI-TOF-MS was used again to monitor the resulting concentrate for the peak of interest.

*SDS-PAGE gels*: Samples were loaded in a Tris-Tricine gel using a buffer containing 50 mM Tris, pH 6.8, 5% glycerol, 1.67% b-mercaptoethanol, 1.67% SDS and 0.0062% bromophenol blue. Protein was boiled for 5 min and resolved independently in 12% acrylamide gels, using a Mini-Protean II electrophoresis cell (Bio-Rad). A constant voltage of 150 V was applied for 45 min for this purpose. The gel was fixed in a solution containing 10% acetic acid, 30% methanol for 30 min, and stained overnight in 0.1% Coomassie Brilliant Blue in 10% acetic acid, 30% methanol. The gels were then washed in a solution containing 10% ethanol and 7% acetic acid for 30 min.

*Tryptic digestion:* Gel bands were first sliced to small pieces, and washed in milli-Q water. Reduction and alkylation was applied by incubation with Ditiothreitol (DTT, 10 mM in 50 mM ammonium bicarbonate, 30 µl) at 56 ºC for 20 min, followed by an incubation in iodoacetamide (50 mM in 50 mM NH_4_HCO_3_, 30 µl) for another 20 min in the dark. Gel pieces were dried and incubated with trypsin (12.5 µg/ml in 50 mM NH_4_HCO_3_, 10 µl) for 20 min in ice. After rehydration, the trypsin supernatant was discarded; spots were covered with 50 mM NH_4_HCO_3_, and incubated overnight at 37ºC. After digestion, acidic peptides were further extracted with trifluoracetic acid 0.1% and dried out in a RVC2 25 Speedvac concentrator (Christ, Osterode Am Harsz, Germany). Peptides were resuspended in 5 µl 0.1% folic acid and sonicated for 5 min. prior to their analysis. Two independent MS/MS methods were followed.

*NanoLC-MS/MS and Data Analysis*: Peptide mixtures obtained from the digestion were separated by on-line nanoLC and analyzed by electrospray tandem mass spectrometry. Peptide separation was performed on a nanoAcquity UPLC system (Waters) connected to an LTQ Orbitrap XL mass spectrometer (Thermo Electron, Bremen, Germany). 2.5 µl of each sample were diluted to a final volume of 5 µl in CH_2_O_2_ 0.1% and loaded onto a Symmetry 300 C18 UPLC Trap column, 180 µm x 20 mm, 5 µm (Waters Corporation, Mildford, MA). The precolumn was connected to a BEH130 C18 column, 75 μm x 200 mm, 1.7 μm (Waters Corporation) equilibrated in 3% acetonitrile and 0.1% HCOOH, and peptides were eluted at 300 nl/min using a 60 min linear gradient of 3–50% acetonitrile directly onto the nanoelectrospray ion source (Proxeon Biosystems, Odense, Denmark).

The mass spectrometer automatically switched between MS and MS/MS acquisition in DDA mode. Survey full scan MS spectra (m/z 400–2000) were acquired in the orbitrap with 30,000 resolution at m/z 400. 6 most intense ions above 1,000 counts were sequentially subjected to CID fragmentation in the linear ion trap. Precursors with charge states of 2 and 3 were specifically selected for collision-induced dissociation. Collision-energy applied to each peptide was automatically normalized as a function of the m/z and charge state. Analyzed peptides were excluded for further analysis during 30 s using dynamic exclusion lists.

Searches were performed using Mascot Search engine (v2.2.07, Matrix Science, London, UK) on Proteome Discoverer 1.2. software (Thermo Electron, Bremen, Germany). Carbamidomethylation of cysteines as fixed modification, oxidation of methionines as variable modification, 5 ppm of peptide mass tolerance, 0.5 Da fragment mass tolerance were adopted as search parameters, and 2 missed cleavages were allowed. Spectra were searched against UniprotKB/Swiss-Prot database (version 2013_01, 539,165 sequences, 191,456,931 residues), restricted to homos sapiens. Only proteins with at least two peptides passing p<0.05 cut-off were considered for protein identification.

*Peptide Mass Fingerprint and Peptide Fragment Fingerprinting:* MALDI-TOF-TOF analysis was performed with a MALDI-LIFT-TOF AUTOFLEX III smartbeam (Bruker Daltonics). Each digested sample was loaded (1 μl) onto a target (Bruker 384 ground steel) with 1μl of α cyano-4-hydroxy-cinamic acid. Data-dependent MS acquisitions were performed with charge state of 1 over a survey m/z range of 500-4000. Ionization was performed with a solid-state laser of 360 nm and 200 Hz. Laser intensity energies were varied depending of the analysis required. For MS 30-50% of intensity was used and around 90% for MS/MS. Resolution was always over 7500 along all mass-window range for MS analysis. Data acquisition was performed manually. Routinely 1400 scans were collected for Peptide Mass Fingerprinting whereas the most intense peaks were selected for MS/MS (400 scans for parent selection, and 1600 scans for fragments). Obtained spectra were processed using Flex analysis 3.0 and Biotools 3.2 (Bruker Daltonics, Billerica, MA). Database searching is performed using Mascot (Matrix Science, London, UK) against UniprotKB/Swiss-Prot database. For protein identification the following parameters were adopted: carbamidomethylation of cysteines (C) as fixed modification and oxidation of methionines (M) as variable modifications, 50 ppm of peptide mass tolerance, 0.7 Da fragment mass tolerance and up to 2 missed cleavage points. Calibration was performed externally, with pepmix (Bruker Daltonics), and internally, with trypsin peptides, when available.
