# Supplementary material for: Lack of a 5.9 kDa Peptide C-Terminal Fragment of Fibrinogen α Chain Precedes Fibrosis Progression in Patients with Liver Disease
Source: PLoS One. 2014 Oct 2;9(10):e109254. doi: 10.1371/journal.pone.0109254 (PMC4183580; doi:10.1371/journal.pone.0109254)
Supplement: Data S3 — Materials and Methods corresponding to the analysis of the messenger RNA expression of human fibrinogen α, β, and γ chains. (DOC) [file pone.0109254.s003.doc]

# EXPANDED EXPERIMENTAL PROCEDURES

***Messenger RNA expression of human fibrinogen α, β, and γ chains in HepG2 cells****:*

Total RNA from cultured cells was extracted using a commercially available kit: Tri Reagent (Molecular Research Center, Inc, Cincinnati, OH). One μg of total RNA was reverse transcribed (RT) by using a complementary DNA synthesis kit (High-Capacity cDNA Reverse Transcription Kit, Applied Biosystems, Foster City, CA). Primers for human fibrinogen α chain (left: 5’-TGGAAATTTTGAGAGGCGATT-3’, right: 5’-CCTCTGACACTCGGTTGTAGG-3’), fibrinogen β chain (left: 5’-CTGAAATGTATCTCATTCAACCTGA-3’, right: 5’- CCGTCTTGACGGTT CTGAAT-3’), fibrinogen γ chain (left: 5’-AGCCCAGCTTGAAGCACA-3’, right: 5’-TTGGCAATGTCTTGACAATCTT-3’), and HPRT (hypoxyxantine-guanine phosphoribosyltransferase 1), a constitutively expressed gene used as an endogenous standard (left: 5’-TGACCTTGATTTATTTTGCATACC-3’, right: 5’- CGAGCAAGACGTTCAGTCCT-3’), were designed according to human fibrinogen α, β, and γ chain and HPRT sequences (GenBank NM_000508.3, NM_005141.4, NM_000509.4, NM_000194.2, respectively) using the ProbeFinder software (Probefinder v 2.49, Roche Diagnostics, Indianapolis, IN). Real time quantitative PCR was analyzed in duplicate and performed with Lightcycler-480 (Roche Diagnostics). Ten µl total volume reaction of diluted 1:8 cDNA, 200 nM primer dilution, 100 nM pre-validated 9-mer probe (Universal ProbeLibrary, Roche Diagnostics) and FastStart TaqMan Probe Master (Roche Diagnostics) was used in each PCR reaction. Fluorescence signal was captured during each of the 45 cycles (denaturizing 10 sec at 95ºC, annealing 15 sec at 60ºC and extension 20 sec at 72ºC). HPRT was used as reference gene for normalization and water was used as negative control. Relative quantification was calculated using the comparative threshold cycle (CT), which is inversely related to the abundance of mRNA transcripts in the initial sample. The mean CT of duplicate measurements was used to calculate ΔCT as the difference in CT for target and reference. Relative quantity of product was expressed as fold-induction of the target gene compared with the control primers according to the formula 2-ΔCT. The mean CT of duplicate measurements was used to calculate ΔCT as the difference in CT for target and reference. The relative quantity of product was expressed as fold-induction of the target gene compared with the reference gene according to the formula 2-ΔΔCT, where ΔΔCT represents ΔCT values normalized with the mean ΔCT of control samples.
